# Challenges in radiobiological modeling: can we decide between LQ and LQ-L models based on reviewed clinical NSCLC treatment outcome data?

**DOI:** 10.1186/s13014-016-0643-5

**Published:** 2016-05-06

**Authors:** Alina Santiago, Steffen Barczyk, Urszula Jelen, Rita Engenhart-Cabillic, Andrea Wittig

**Affiliations:** Department of Radiotherapy and Radiation Oncology, University Hospital Giessen and Marburg, Philipps-University Marburg, Baldingerstrasse, Marburg, 35043 Germany; Present address: Gemeinschaftspraxis Strahlentherapie am St. Agnes Hospital, Bocholt, Germany; Present address: Marburger Ionenstrahl-Therapiezentrum MIT, Marburg, Germany

**Keywords:** Non-small cell lung cancer, Dose-response modeling, Biologically effective dose, Linear-quadratic model, Alpha-beta ratio

## Abstract

**Aim:**

To study the dose-response of stage I non-small-cell lung cancer (NSCLC) in terms of long-term local tumor control (LC) after conventional and hypofractionated photon radiotherapy, modeled with the linear-quadratic (LQ) and linear-quadratic-linear (LQ-L) approaches and to estimate the clinical α/β ratio within the LQ frame.

**Material and methods:**

We identified studies of curative radiotherapy as single treatment through MedLine search reporting 3-year LC as primary outcome of interest. Logistic models coupled with the biologically effective dose (BED) at isocenter and PTV edge according to both the LQ and LQ-L models with α/β = 10 Gy were fitted. Additionally, α/β was estimated from direct LQ fits.

**Results:**

Thirty one studies were included reporting outcome of 2319 patients. The LQ-L fit yielded a significant value of 11.0 ± 5.2 Gy for the dose threshold (D_t_) for BED_10_ at the isocenter. The LQ and LQ-L fits did not differ substantially. Concerning the estimation of α/β, the value obtained from the direct LQ fit for the complete fractionation range was 3.9 [68 % CI: 2.2–9.0] Gy (*p* > 0.05).

**Conclusion:**

Both LQ and LQ-L fits can model local tumor control after conventionally and hypofractionated irradiation and are robust methods for predicting clinical effects. The observed dose-effect for local control in NSCLC is weaker at high doses due to data dispersion. For BED_10_ values of 100–150 Gy in ≥3 fractions, the differences in isoeffects predicted by both models can be neglected.

**Electronic supplementary material:**

The online version of this article (doi:10.1186/s13014-016-0643-5) contains supplementary material, which is available to authorized users.

## Introduction

The linear-quadratic (LQ) model was developed to describe experimental survival curves of both normal and tumor cells after irradiation. The LQ model fits the cell surviving fraction through a second-order polynomial on the dose per fraction, with coefficients α and β. The ratio between both coefficients describes the repair capacity of the cells and thus sensitivity to fractionation [1, 2].

The LQ model provides an accurate description of fractionation effects at doses between 1 and 8–10 Gy per fraction [3]. Essentially, this formalism enables isoeffect calculations in current clinical practice, defining the relationships between the biological irradiation effect and key parameters such as dose per fraction, total number of fractions and treatment time. Advancements in this model led to the two most extended, complementary approaches for isoeffect calculation: the biologically effective dose (BED) and the equivalent dose in 2 Gy per fraction (EQD2) [4].

Current treatment of choice for stage I non-small cell lung cancer (NSCLC) is surgical tumor extraction. Since it became technically feasible, radiotherapy has been used as an alternative treatment method in inoperable cases. Early approaches used 3D-conformal conventionally fractionated techniques, whereas today, stereotactic body radiotherapy (SBRT) allows for highly precise delivery of radiation, thus enabling hypofractionation to deliver ablative radiation doses in 1–5 fractions [5]. Therefore, SBRT evolved to be the current treatment of choice for early-stage NSCLC in medically inoperable patients and in patients who do not consent to surgery. The high precision of dose delivery facilitates normal tissue sparing, even allowing for dose escalation to potentially improve local control. Despite a growing pool of clinical outcome data, the optimal total dose and fractionation scheme to reach the intended biological effects in terms of both local tumor control and side effects are still under debate [6].

Hypofractionation requires reliable isoeffect calculations. Thus, the relevance of the question has been renewed, whether the description of radiobiological effects based on the LQ formalism is appropriate for hypofractionated treatments [7–9].

If no α/β ratio estimation is available for a specific tumor entity, a generic value of 10 Gy is used for BED calculations, although the precision achievable with such a standard α/β ratio is assumed to be lower. Numerous attempts to calculate clinical α/β values have been made, using available clinical outcome data [10]. Such estimations are specially needed if the α/β of a specific tumor entity is suspected to be lower than 10 Gy. In this case a modified fractionation scheme, which reduces tumor cell recovery between fractions, could increase the therapeutic ratio as is the case e.g. in prostate carcinoma or breast cancer.

Many recent studies aim at outcome review and modeling of the dose response relationship of NSCLC [11–15]. However, studies attempting to estimate the α/β ratio for NSCLC are scarce [11, 15, 16]. It is subject of current debate if the improved outcomes of hypofractionated SBRT are a consequence of an α/β ratio lower than 10 Gy, or even lower than the α/β value of the surrounding normal tissue, which could add a radiobiological rationale to the use of hypofractionation. Alternatively, the improvement could be caused by a reduced repopulation in a shorter overall treatment time.

In addition, in particle radiotherapy, a currently emerging field in radiation oncology, radiobiological considerations are of importance. For proton radiotherapy hypofractionated concepts are aimed for partially as motion management strategy [17–20], so that isoeffect calculations are essential. Apart from isoeffect calculations current treatment planning strategies for light ion therapy also require the attribution of radiobiological properties to both tumor and normal tissues. Specifically, in scanned-beam carbon ion therapy, radiosensitivity is characterized through α/β values obtained from photon irradiation experiments in vitro [21] in one of the mathematical models describing the enhanced biological effect in the Bragg peak, the so called local effect model (LEM), which is implemented in commercial treatment planning systems.

In such situation, clinical long time follow up data is the most valid source of data for modeling approaches, which is however inherently limited by inhomogeneity of treatment parameters and treatment techniques evolving over time. We focused our analysis on the comparison of the LQ-L versus the LQ model, since most of the mathematical model corrections to the LQ model proposed need additional input parameters [16, 22–24], which are not available for the specific clinical situation.

Therefore, this work aims at:

1. investigating the dose-response of NSCLC tumor control data from conventionally fractionated (CF) and stereotactic, hypofractionated radiotherapy treatments (HF), based on a review of published long-term outcome results,
2. evaluating the validity of the LQ and LQ-L models for both conventional and SBRT treatments,
3. and obtaining an estimation of the clinical α/β ratio of NSCLC.

## Materials and methods

### Study design

We identified inclusion criteria, search strategy, outcome measures of interest and indispensable treatment parameters for the study. The analysis keeps standards of the Preferred Reporting Items for Systematic Reviews and Meta-Analyses (PRISMA) statements [25].

### Selection criteria

PubMed was searched (July 2015) without restrictions on the publication date. Abstracts of conference proceedings were excluded and language was restricted to English. Repeated publications based on the same cohort were excluded as were outcome reviews in order to avoid duplicity of cohorts. The selection of studies based on the following criteria was made by two independent researchers.

Study cohorts were eligible only if the following criteria were fulfilled:

1. Patients with stage I NSCLC (cT1/2, cN0, cM0) with either central or peripheral tumor location.
2. Treatment with photon radiotherapy with curative intent either 2D or 3D-conformal radiotherapy or SBRT as single modality treatment. Treatment could be delivered with CyberKnife, GammaKnife or linac-based without restrictions on fractionation schemes (e.g. normofractionated, accelerated, hyper- or hypofractionated schedules, single fraction irradiation), provided complete information on number of fractions, dose per fraction, total absorbed dose and overall treatment time was available.
3. Reported outcome of interest (actuarial 3-year local control estimations according to Kaplan-Meier or other methods, in which death was a censored event) with a median follow-up time in each cohort ≥17 months.
4. From an original study (i.a. prospective randomized controlled trial or prospective or retrospective observational study or case series).
5. The reported cohorts must include at least 25 patients.
6. Patients could be judged to be either operable or inoperable if the clinical decision was made in favor of radiotherapy.
7. A small amount of heterogeneity in reported dose and patient parameters for a given outcome data point was accepted (e.g. a marginal group of patients treated with a deviating radiation scheme, or a small proportion of tumors included in the report of a stage I population with clinical staging other than I).

### Data extraction

The main endpoint of this review was local tumor control (LC) at 3 years. If this value was not explicitly provided in the text, it was extracted from the Kaplan-Meier diagrams.

The fractionation concept (dose per fraction, number of fractions and total treatment time), and the planning technique were extracted together with further treatment- and patient-related parameters. The range of variation in reported cohort parameters for each local tumor control data point was qualitatively assessed and highly heterogeneous cohorts were excluded. Reported mean or preferably median dose values were used to describe the outcome of each specific patient cohort. All prescription doses were translated to doses at the isocenter and at PTV edge, calculated according to the information provided in each publication (more details in the Additional file 1). Mathematical modeling was performed with both, doses at the isocenter and at the PTV edge. The treatments were classified to be hypofractionated (HF) if 1–10 fractions were delivered with doses per fraction at the isocenter above 6 Gy. Treatments were classified as conventionally fractionated (CF) with a broader definition than in the clinical convention, based on the validity limits of the LQ model according to our current knowledge, namely treatment delivered in more than 10 fractions with fraction doses at isocenter ranging between 1.2 and 6 Gy.

### Data analysis and mathematical models

Model parameters were fitted with nonlinear least square optimization methods and confidence intervals were calculated with likelihood profiling. A logistic relationship between tumor control probability (TCP) and the biological effective dose (BED) was assumed, according to the parameterization described in Okunieff et al. [26] and Bentzen et al. [27]. BED was based on the LQ model, calculated from the number of fractions and the dose at the isocenter, taking into account neither repopulation nor hypoxia, according to Eq. 1:

1 $$ TCP=\frac{exp\left[\left( BE{D}_{LQ}-TC{D}_{50}\right)/k\right]}{1+ exp\left[\left( BE{D}_{LQ}-TC{D}_{50}\right)/k\right]}, with\; BE{D}_{LQ}=nd\left(1+\frac{d}{\alpha /\beta}\right) $$

where TCD_50_ is the dose necessary to obtain a local tumor control of 50 % and k is a parameter with dose units that is used to calculate the normalized slope, γ_50_. This parameter quantifies the change in the expected TCP when a 1 % change in dose occurs, evaluated at the dose level of the TCD_50_, and represents the maximal slope of the dose-response relationship. It can be calculated from k and TCD_50_ with the expression [27, 28]:

2 $$ {\gamma}_{50}=\frac{4k}{TC{D}_{50}} $$

The same logistic model was implemented with an alternative BED definition, including a transition from linear-quadratic dependence for the cell survival to purely linear beyond a certain dose level, the dose threshold D_t_, as described in [22]:

3 $$ BE{D}_{LQ-L}=\left\{\begin{array}{l}nd\left(1+\frac{d}{\alpha /\beta}\right)\kern10em for\;d<{D}_t,\\ {}n{D}_t\left(1+\frac{D_t}{\alpha /\beta}\right)+n\left(\frac{\alpha +2\beta {D}_t}{\alpha}\right)\left(d-{D}_t\right)\kern1em for\;d\ge {D}_t,\end{array}\right\} $$

where n is the number of irradiation fractions, d is the fraction dose, and D_t_ is the threshold for the fraction dose.

The LQ and LQ-L models were fitted to the joint dataset, and to the CF and HF subsets separately, for BED doses calculated both at the isocenter and the PTV edge. First, the linear-quadratic (LQ) model was applied with α/β fixed to 10 Gy, as it is universally accepted for conventional fractionation. The alternative BED definition derived from the LQ-L model was also applied with α/β equal to 10 Gy, to test if the inclusion of a dose threshold D_t_ would improve the previous fit. Additionally, a study to tentatively estimate the α/β ratio from these clinical data was carried out.

In summary, the following fits were calculated:

1. LQ model with α/β ratio fixed to 10 Gy on the full dataset, and on the HF and CF datasets separately.
2. LQ-L model with α/β ratio fixed to 10 Gy on the full and on the HF datasets.
3. LQ model with free γ_50_, TCD_50_ and α/β ratio on the full and the CF datasets.

Finally, in order to compare with the results of the recent analysis of Chi et al. [15] the Spearman’s correlation between 3-year LC and BED calculated with different fixed α/β ratios was also investigated on the full and on the HF datasets.

The quality of the fits was assessed with checks of the residuals for normality. Models were ranked according to the Akaike Information Criterion (AIC), and for the LQ and LQ-L, being nested models, maximum likelihood ratio tests were made. All fitted and calculated values are reported together with their 68 % confidence intervals (CI), whenever possible. Statistical significance was assumed for *p* values < 0.05. Data handling, statistical analysis, model fitting and graphing were done with the software package R, version 2.15.0 [29].

## Results

### Selected patient cohorts and description of the studies

In total, 31 studies were identified, which fulfilled the selection criteria, Of those, 8 studies report outcomes after conventionally fractionated treatments of a total of 344 patients [30–37] and 23 studies including 1975 patients reporting on hypofractionated irradiations [38–60]. A total of 34 local control - schedule data points, with doses per fraction ranging from 30 to 1.2 Gy, applied in 1–58 fractions, were collected (see Tables 1 and 2, and Additional file 1 for details of the publication search).

**Table 1:** Characteristics of included studies with conventionally fractionated treatment regimes. Studies published between 1993 and 2015

No.	Reference	No. pats.	No. of pats. with stage T1 - T2	Fractionation regime			BED_10_@ isoc [Gy]	BED_10_@ PTV edge [Gy]	Dose calculation algorithm	3y-LC [%]	Follow-up Median (range) [m]
				D [Gy]	d [Gy]	T [d]					
1	Kaskowitz 1993 [30]	53	20–33	63 (40–80)	conventional	ns	74.3	69.6	ns	51	ns
2	Jeremic 1997 [31]	49	25–24	69.6	1.2 (2× day)	40	78.0	70.8	ns	55	ns
3	Hayakawa 1999 [32]	36	7–29	60–81	2	48	80.4	75.7	no dens corr	72	(36–216)
4	Cheung 2002 [33]	33	18–15	48	4	21	67.2	62.9	dens corr	63	23
5	Langendijk 2002 [34]	46	26–20	70	2	49	84.0	79.1	dens corr	50	36
6	Bradley 2003 [35]	56	31–25	60–84	1.8–2	42–56	83.7	78.6	no dens corr	63	20 (6–72)
7	Bogart 2005	31	19–12	70	2.3–3.7	39	87.5	83.3	ns	83	29
8	Zehentmayr 2015 [36]	40	19 (Ia)–21 (Ib)	79.2 (73.8–90)	1.8 (2× day)	30–42	93.5	87.5	ns	91	28.5 (2–108)
	Median	43		69.8	2.0	44	82.0	77.2		63	28.5 (2–216)

*BED*_*10*_ biologically effective dose with α/β = 10 Gy, *PTV* planning target volume, *D* total dose, *d* dose per fraction, *T* total treatment time, *LC* local control, *ns* not specified

**Table 2:** Characteristics of included studies with hypofractionated treatment regimes. Studies published between 2003 and 2015

No.	Reference	No. pats.	No. of pats. with stage T1 - T2	Fractionation regime			BED_10_@isoc [Gy]	BED_10_@PTV edge [Gy]	Dose calculation algorithm	3y-LC [%]	Follow-up median (range) [m]
				D [Gy]	d [Gy]	T [d]					
9	Onimaru 2003 [38]	25	17–8	48/60	6/7.5	14	76.8	56.8	dens inhom corr	55	18 (2–44)
10	Xia 2006 [39]	25	ns	50	5	^14^	200	75	GammaKnife, ns	96	27 (24–54)
11	Fritz 2008 [40]	40	22–18	30	30	1	120	81.6	modified Batho	81	20 (6–62)
12	Onimaru 2008 [41]	41	13–28	40/48	10/12	5	105.6	75.3	ns	57	27 (9–62)
13	Baumann 2009 [42]	57	40–17	45	15	5 (4–15)	211.2	112.5	PB, dens inhom corr	92	35 (4–47)
14	Brown 2009 [43]	31	20–11	60–67.5	3–5	5	347.5	180.0	ns	86	28 (24–53)
15	Fakiris 2009 [44]	70	34–36	60/66	20/22	5	309.4	211.2	no dens inhom corr	88	50 (1–65)
16	Kopek 2009 [45]	88	51–36	45/67.5	15/22.5	5–8	112.5	60.9	Helax-TMS/ Eclipse, ns	89	44 (2–97)
17	Stephans 2009 [46]	56	42–14	50	10	11 (8–14)	168	100.0	dens inhom corr	97	20 (2–48)
18	Baba 2010 [47]	124	87–37	48/52	12/13	11	105.6/119.6	75.3/84.9	PB convol with Batho	80	26 (7–66) (living pats)
19	Crabtree 2010 [48]	76	57–19	54	18	8–14	219.4	151.2	Trilogy, ns	89	19
20	Timmerman 2010 [49]	55	44–11	54	18	14	286.4	151.2	dens inhom corr	98	34 (5–50)
21	Videtic 2010 [50]	26	22–6	50	10	5	112.3	100	dens inhom corr	94	31 (10–51)
22	Andratschke 2011 [51]	92	31–61	24/45	3/5	5–12	192.2	84.4	dens inhom corr	83	21 (3–87)
23	Hamamoto 2012 [52]	128	101–27	48/60	9.2–14	4–10	105.6	89.9	PB, no dens inhom corr	85	18 (1–60)
24	Lagerwaard 2012 [53]	177	106–71	60	12 20 7.5	14	187.5	132.0	Brainlab, ns	93	32
	Shibamoto 2012 [54]							75.3	PB convo, Batho	83	36
25a	Shibamoto, d2	124	124 T1	48	12	9–21	105.6	75.3		86	
25b	Shibamoto, d3	52	52 T2	52	13	9–21	119.6	84.9		73	
	Shirata 2012 [55]		63–18					89.9	PB convolution Batho	89	30 (0.3–79)
26a	Shirata, d1	45		48	12		105.6	89.9		100	
26b	Shirata, d2	29		60	7.5		105	91.4		82	
	Takeda 2012 [56]								XiO/CMS, CS		
27a	Takeda, d1	27	10–17	40	8	5	100	72.0		72	21 (6–64)
27b	Takeda, d2	138	91–47	50	10	5	140.6	100.0		87	21 (6–64)
28	Inoue 2013 [57]	109	79–30	45/48	15/12	4–7	105.6	75.3	dens inhom corr	81	25 (4–72)
29	Takeda 2013 [58]	109	67–42	40/50	8/10	5	140.6	100	convolution-superposition	84.4	24 (3–65)
30	Hamaji 2015 [59]	104	75–29	48	12	5	105.6	75.3	PB convol, Batho	76.7	43 (6–115)
31	Rwigema [60]	46	-	54	18	5	234.5	151.2	MC	95.5	16.8 (0.6–38.9)
	Median	57		56.0	12.5	7	119.8	89.9		86	27.0 (0.3–115)

*BED*_*10*_ biologically effective dose with α/β = 10 Gy, *PTV* planning target volume, *D* total dose, *d* dose per fraction, *T* total treatment time, *LC* local control, *ns* not specified, *dens inhom corr* density inhomogeneity correction, *PB convol* pencil beam convolution, *CS* convolution superposition

Of all reported tumors, 63.6 % were confirmed to be stage T1, 36.4 % T2 (Table 3). A total of 68.1 % of tumors were histologically confirmed: 45.8 % adenocarcinomas, 34.1 % squamous cell carcinomas, 6.2 % other histologies and 13.9 % carcinoma not otherwise specified (NOS). Of the patients treated with conventional fractionation 86.3% were confirmed medically inoperable, versus 55.2 % of all patients treated with hypofractionated schedules. Median of the reported median ages [age range] was comparable between both groups, namely 72 [range: 35–90] and 75 [range: 29–94] years in the CF and HF groups respectively. Patients, who received conventionally fractionated RT were treated in the time period from 1976 to 2010, whereas patients treated with hypofractionated regimes were irradiated in the time period from 1996 to 2012. In the CF cohort only in one study PET-CT was performed for staging in 6 out of 31 patients (Bogart et al. [36]), whereas for many of the HF cohorts PET was a routine procedure; for many of the most recent studies PET-staging was even an inclusion criterion in the retrospective series.

**Table 3:** Summary of cohort characteristics and clinical follow up for conventionally fractionated and hypofractionated datasets

Dataset	Total No. pats.	% T1	Histology				% histology unknown	% inoperable	Median age (range) [y]	Median follow-up (range) [m]	Median BED_10_@isoc (range) [Gy]	Median No. Pats (range)
			% Adeno	% SCC	% NOS	% Other						
CF	344	48.0 %	26.3	47.0	15.5	11.2	11.6	86.3	72 (35–90)	28.5 (2–216)	82.0 (67.2-93.5)	43 (31–56)
HF	1975	66.3 %	50.4	31.1	13.5	5.0	35.9	55.2	75 (29–94)	27 (0.3–115)	119.6 (76.8–347.5)	57 (25–177)
Total	2319	63.6 %	45.8	34.1	13.9	6.2	31.9	59.8	74 (29–94)	27 (0.3–216)	105.6 (67.2–347.5)	53 (25–177)

*Adeno* adenocarcinoma, *SCC* squamous cell carcinoma, *NOS* carcinoma not otherwise specified, *HF* hypofractionated treatment regime, *CF* conventionally fractionated treatment schedule

In the 8 series of the CF group, generally a margin of 1–1.5 cm was added around the gross tumor volume (GTV), which was in some cases estimated from port films if no planning computer tomography (CT) scan was available. In the HF series, most frequently no GTV-to-CTV (clinical target volume) margins were added, except in 5 out of 23 series. Internal target volume (ITV) concepts were applied in 13/23 studies, based either on addition of GTV from 3D-CT scans in expiration/inspiration and free breathing in 4 cases, on slow CT scans in 5 cases, and on 4D-CT scans in 4 cases. Nine out of 23 studies did not apply any ITV concept. The most frequently used CTV-to-PTV (planning target volume) margins were 0.5 cm in axial and 1 cm in cranio-caudal directions. A minimal margin of 0.2 cm was added in one patient cohort treated with the CyberKnife where tumor tracking was used to correct for intrafractional target motion. In 2 out of 23 series the PTV margin definition was patient-specific.

Different dose reporting concepts were found throughout the selected references. Only 5 CF reports out of 8 explicitly mentioned that the dose was prescribed to the isocenter. When not specified, prescription to the isocenter was assumed. In the case of the hypofractionated SBRT data, 9 references reported the prescribed dose to isocenter and 11 to the isodose line encompassing the PTV, which ranged from the 50 to the 100 % isodose, most frequently to the 80 % isodose line. Only one of the SBRT cohorts was treated with IMRT, and in this case the dose was prescribed to the 95 % isodose line enclosing the PTV. More information can be found in the Additional file 1.

Median [min - max] applied BED at the isocenter, calculated with an α/β ratio of 10 Gy (BED_10_), was 82.0 [67.2–93.5] and 119.8 [76.8–347.5] Gy for the CF and HF groups, respectively. At the PTV edge, values of 77.2 [62.9–87.5] and 89.9 [56.8–211.2] Gy were applied in the CF and HF groups.

Median value of the documented median follow-up times were 28.5 and 27 months for the CF and HF groups, respectively. Very few of these publications state explicitly the number of patients at risk at each follow-up time point (four cohorts). In only 5 of the selected studies an estimation of the 95 % CI of the calculated actuarial local control rates was reported and one publication presents the standard error. Therefore, no information could be collected about the precision of the estimated actuarial local control rates, apart from the cohort size and the median follow-up time.

### 3- and 5- year clinical outcomes for stage I NSCLC

The median value [range] for the 3-year LC was 86 [range: 55–100] % for the HF, and 63 [range: 50–91] %, for the CF series, respectively. Twenty-two out of 26 HF 3-year LC data points lie above the 80 % level, while all except two of the CF datasets lie below. All except one HF treatments delivered a BED_10_ of 100 Gy or higher at the isocenter but none of the CF treatments reached a BED_10_ of 100 Gy.

Spearman’s correlation coefficients between dosimetric parameters (total absorbed dose and BED_10_) and the different clinical outcomes were calculated. For the complete dataset and the total BED_10_ at the isocenter, a significant Spearman’s correlation of 0.716 for the local control with BED_10_ was found. For the BED_10_ evaluated at the PTV edge, a significant correlation of 0.638 was found between LC and BED_10._ The total absorbed dose at either dose point however did not correlate with LC.

### Modeling local control versus BED

#### Linear-quadratic (LQ) model with α/β ratios fixed to 10 Gy (two-parameter fit)

**Fig. 1:**
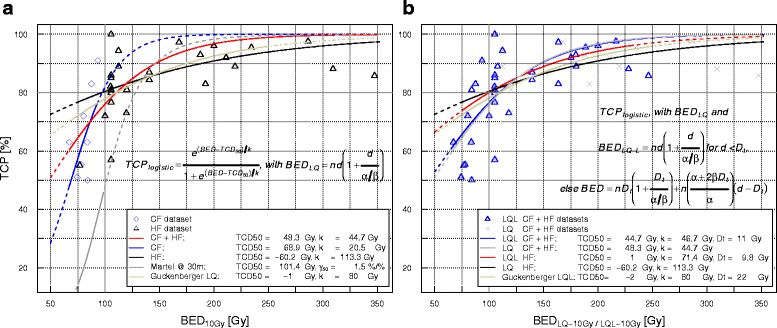
**a** Clinical 3-year LC data points for the conventionally fracionated and hypofractionated datasets versus BED_10_ at isocenter with corresponding logistic regression fits of the joint CF + HF dataset, and the CF and HF subsets. Previously published related models are included for comparison: Martel et al. [28] and Guckenberger et al. [61]. **b** Comparison between the BED_10_ (isocenter) data points obtained with the LQ and LQ-L fits for the complete dataset and for the hypofractionated subset. The range of experimental data of every model is shown with a continuous line and the extrapolation regions with dashed lines. The levels 80 % TCP and 100 Gy BED_10_ are also shown for reference

All 3-year LC data points for both the HF and CF treatments were fitted to a logistic model coupled with BED_10_ at both isocenter and PTV edge. For the isocenter, the TCD_50_ [68% CI] was 48.3 [23.8–62.4] Gy, k was 44.7 [32.1–64.8] Gy, and the calculated γ_50_ (std error) was 0.27 ± 0.1. A logistic fit under the same assumptions, based on the CF subset alone was made, and resulted in the values: TCD_50_ of 68.9 [50.7–74.4] Gy, k was 20.5 [13.1–50.0] Gy and γ_50_ was 0.84 ± 0.5. The same approach applied to the HF dataset showed a TCD_50_ of −60.2 [−189–3.2] Gy, k of 113.3 [73.4–190.1] Gy, and γ_50_ of −0.13 ± 0.17. These three logistic models are represented in Fig. 1a, and all fit parameter values are summarized in Table 5. The *p* value was found to be < 0.05 for both TCD_50_ and k simultaneously only for the model based on the full dataset.

**Fig. 2:**
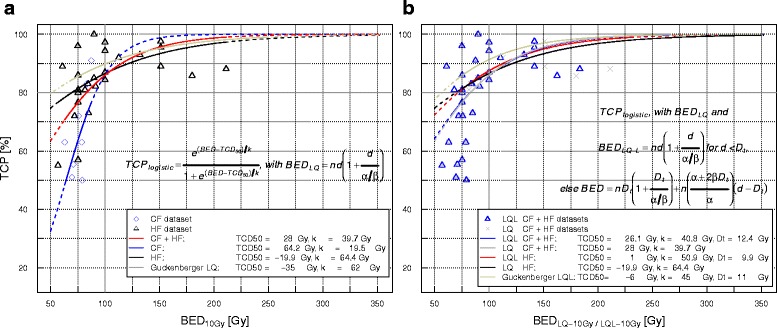
**a** Clinical 3-year LC data points for the CF and HF datasets versus BED_10_ at PTV edge, with corresponding logistic regression fits of the joint conventionally and hypofractionated dataset, and the conventionally fractionated and hypofractionated subsets. A previously published related model is included for comparison, Guckenberger et al. [61]. **b** Comparison between the BED_10_ (PTV edge) data points obtained with the LQ and LQ-L fits for the complete dataset and for the hypofractionated subset. The range of experimental data of every model is shown with a continuous line and the extrapolation regions with dashed lines. The levels 80% TCP and 100 Gy BED_10_ are also shown for reference

The model for the complete dataset based on BED_10_ at PTV edge yielded a TCD_50_ of 28.0 [−0.7–43.1] Gy, and k was equal to 39.7 [28.1–60.5] Gy. These values were 64.2 [48.6–69.3] and 19.5 [12.5–44.6] Gy respectively for the CF, and −19.9 [−93.5–14.5] and 64.4 [42.9–108.1] Gy for the HF group alone. Only the parameter values for the CF dataset yielded significant *p* values simultaneously. Model summaries and calculated γ_50_ values together with their standard errors obtained with propagation of uncertainties are also presented in Tables 5. These models are represented in Fig. 2a.

#### Linear-quadratic-linear (LQ-L) model with α/β fixed to 10 Gy (three-parameter fit)

The alternative BED definition including a linear portion in the dose-effect beyond a certain fraction dose was tested, according to Eq. 3, with an α/β ratio fixed to 10 Gy. Fits were performed either based on the full dataset or on the HF subset alone.

For the models based on BED_10_ at the isocenter, the fit based on the full dataset generated a D_t_ value of 11.0 [8.4–16.7] Gy, together with a TCD_50_ value of 44.7 [24.2–58.2] Gy, and k equal to 46.7 [35.1–63.2] Gy. The calculated γ_50_ value was 0.24 ± 0.11. All of the parameter estimates yielded significant *p* values. 13 out of 34 data points represented a dose per fraction below the estimated D_t_. This fit is shown in Fig. 1b, together with the LQ fit for comparison, and their respective BED_LQ_ and BED_LQ-L_ data points.

For the fit based on the HF dataset alone, D_t_ was equal to 9.8 [5.2–15.0] Gy, TCD_50_ was 1.0 [na-23.8] Gy, and k equal to 71.4 [54.2–79.6] Gy. Only two data points were found to be below this D_t_, which was too few to obtain a reliable estimate. Thus, the fit parameters did not produce significant *p* values. This fit is shown in Fig. 2b, together with the LQ fit for comparison.

When BED_10_ doses at PTV edge were used, similar D_t_ values were found, namely, 12.4 Gy for the complete dataset, and 9.9 Gy for the HF dataset (this fit is shown in Fig. 2b). Likelihood ratio tests showed no difference between LQ-L and LQ fits, independently of which BED_10_ doses were used, at isocenter or PTV edge (see Table 5).

#### Correlation of local control with BED

To complete the modeling study, the correlation of the 3-year LC with BED under different assumptions for α/β equal to 5, 8.6, 10, 15 and 20 Gy was investigated (see Table 4). For the complete dataset and BED_10_ values at isocenter, a Spearman’s correlation of 0.716 (*p* < 0.0001) with BED_10_ was found. For all other α/β ratios, correlation values increased marginally from BED_5_ (*r* = 0.706) to a maximum at BED_10_ and decreased again for BED_20_ (*r* = 0.706), in all cases being significant. The Spearman’s correlations based on the BED values at the PTV edge decreased with growing α/β values for the complete dataset, from 0.680 to 0.510, all of them being significant and consistently lower than the respective values for the BED_10_ at the isocenter (see Table 4).

**Table 4:** Spearman’s correlations of the local control with the biologically effective dose at the isocenter and the planning target volume edge, calculated with different α/β values, for the complete dataset and exclusively for the hypofractionated dataset (all of them significant, *p* > 0.05)

Spearman’s r	vs BED @ Isocenter		vs BED @ PTV edge	
α/β [Gy]	All datasets	HF	All datasets	HF
5	0.706	0.575	0.680	0.531
8.6	0.716	0.587	0.680	0.560
10	0.716	0.587	0.638	0.542
15	0.749	0.601	0.572	0.606
20	0.706	0.618	0.510	0.601

*BED* biologically effective dose, *PTV* planning target volume

In contrast, for the HF subset, the correlation of the LC with the same series of BED_α/β_ at isocenter increased minimally from 0.575 to 0.618, and also the values for BED_α/β_ at PTV edge, from 0.531 to 0.601, all of them being statistically significant.

#### LQ model with three-parameter logistic fit

**Fig. 3:**
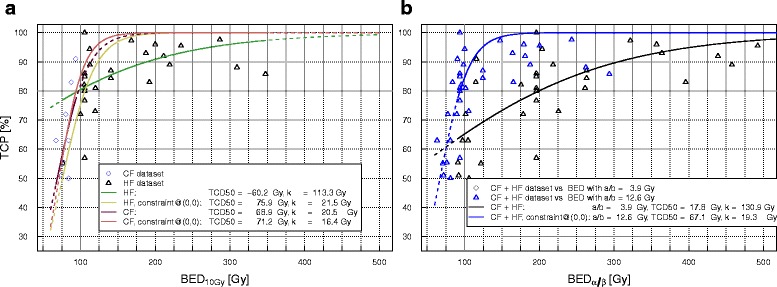
Logistic fits for LC versus BED_α/β_ at isocenter, with constraint to approach the sigmoidal curves to the coordinate origin, **a** with α/β fixed to 10 Gy for the CF and HF datasets separately, and **b** with three free parameters: α/β, TCD_50_ and k, for the combined dataset with conventionally fractionated and hypofractionated treatments. Logistic fits without constraint are provided for comparison

Additionally, we attempted to estimate the α/β ratio for NSCLC from the complete data set, fitting all three model parameters simultaneously: TCD_50_, k and α/β. We obtained for the BED doses at isocenter an α/β value of 3.9 [2.2–9.0] Gy, TCD_50_ of 17.8 [na-56.4] Gy, and k of 130.9 [50.1-na] Gy, with only TCD_50_ yielding a *p* value < 0.05. For the CF dataset alone we found a similar α/β of 3.8; however, it was not possible to determine confidence intervals. These fits are represented in Fig. 3b. In order to check plausibility of the fit results and to compare our results with published values we calculated γ_50_ and found a value of 0.0 ± 0.15. For the models based on BED_10_ at PTV edge we found values for alpha/beta of 1.7 [1.3–4.1] Gy for the complete dataset and 4.1 [na] Gy for the CF dataset with no significant *p* values (Table 5).

**Table 5:** Summary of the models for both BED doses calculated at isocenter and PTV edge; all fit parameter values are provided with standard errors (and 68 % CI). This table includes the maximum likelihood ratio tests for comparison between the corresponding LQ and LQ-L models with α/β = 10 Gy

Model concept and dataset		α/β (std error) [Gy]	D_t_ (std error) [Gy]	TCD_50_CI 68 % [Gy]	k CI 68 % [Gy]	γ_50_(std error) [%/%]	AIC	Likelihood ratio test LQ vs LQL: Dataset, Df, LogLik, Df, Chisq, Pr (>Chisq)
ISOCENTER								
LQ fixed α/β	CF + HF	α/β = 10	-	48.3 (23.8–62.4)*	44.7 (32.1–64.8)*	0.27 (0.1)	−4438.1	Isocenter, all data 4, 2223.8, 1, 3.42, 0.064
	CF	α/β = 10	-	68.9 (50.7–74.4)*	20.5 (13.1–50.0)	0.84 (0.5)	−186.4	-
	HF	α/β = 10	-	−60.2 (−189–3.2)	113.3 (73.4–190.1)*	−0.13 (−0.17)	−2625.2	Isocenter, HF 4, 1316.4, 1, 1.68, 0.195
LQ-L fixed α/β	CF + HF	α/β = 10	11 (8.4–16.7)*	44.7 (24.2–58.2)*	46.7 (35.1–63.2)*	0.24 (0.11)	−4439.6	
	HF	α/β = 10	9.8 (5.2–15.0)	1.0 (na–23.8)	71.4 (54.2–79.6)	0.0 (0.15)	−2624.8	
LQ: free α/β, TCD50 and k	CF+ HF	3.9 (2.2–9.0)	-	17.8 (na–56.4)	130.9 (50.1–na)	0.0 (0.15)	−4441.6	
	CF	3.8 (na)	-	90.7 (na)*	19.7 (na)	1.15 (0.64)	−186.6	
PTV EDGE								
LQ fixed α/β	CF + HF	α/β = 10	-	28.0 (−0.7–43.1)	39.7 (28.1–60.5)*	0.18 (0.13)	−4430.1	PTV Edge, all data 4, 2218.3, 1, 0.614, 0.433
	CF	α/β = 10	-	64.2 (48.6–69.3)*	19.5 (12.5–44.6)*	0.82 (0.47)	−186.6	
	HF	α/β = 10	-	−19.9 (−93.5–14.5)	64.4 (42.9–108.1)*	−0.08 (−0.16)	−2625.9	PTV Edge, HF 4, 1315.9, 1, 0.009, 0.925
LQ-L fixed α/β	CF + HF	α/β = 10	12.4 (8.3–na)	26.1 (na)	40.8 (na–60.2)	0.16 (0.13)	−4428.7	
	HF	α/β = 10	9.9 (5.5-na)	1 (na–18.0)	50.9 (40.0–55.3)	0 (0.15)	−2623.9	
LQ: free α/β, TCD50 and k	CF+ HF	1.7 (1.3–4.1)	-	29.8 (na–68.2)	161.1 (60.4–na)	0.0 (0.13)	−4443.5	
	CF	4.1 (na)	-	81 (na)	20.4 (na)	0.99 (na)	−186	

*D*_*t*_ dose threshold, *TCD*_*50*_ tumor control dose 50 %, *AIC* Akaike information criterion, *Df* degrees of freedom, *LogLik* log-likelihood, *Chisq* chi-square, *PTV* planning target volume

*p value < 0.05

#### Differences in the prediction of isoeffects

**Fig. 4:**
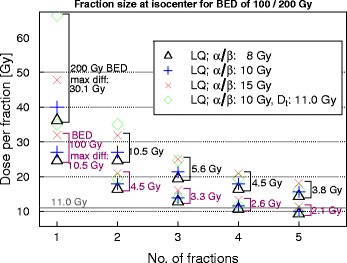
Fraction dose necessary to deliver a BED equal to 100 and 200 Gy under the assumptions of the LQ model with α/β of 8, 10, and 15 Gy and LQ-L model with α/β equal to 10 Gy and a D_t_ value of 11.0 Gy, estimated for doses at the isocenter

In order to translate the impact of the different BED model assumptions on clinical treatment schedules we calculated as an example the doses per fraction which would be necessary to reach selected BED_10_ levels at the isocenter (Fig. 4). Under the assumptions of the LQ model with α/β values of 8, 10, or 15 Gy and the LQ-L model at the isocenter with α/β equal to 10 Gy and a D_t_ of 11.0 Gy, in order to reach 100 Gy (BED), the maximum differences among models for one and two fractions are 10.5 and 4.5 Gy, respectively. Maximal differences remain below 3.3 Gy for treatments delivered in 3 or more fractions. For 200 Gy (BED), discrepancies between models in fraction size increase to 30.1 and 10.5 Gy for one and two fractions, respectively and remain below 5.6 Gy for 3 fractions and more.

## Discussion

Review of clinical outcome data after radiotherapy treatment represents the only possibility to gather long-term information from large numbers of patients, which could serve as basis for statistical analysis for radiobiological modeling. However, this task presents a number of challenges since these datasets are intrinsically heterogeneous. Variability among radiotherapy centers applies to aspects such as target volume definition, dose prescription, planning concepts and delivery techniques with different precision levels. Additional effects hindering precise dose-effect modeling are: the use of multiple and sometimes less appropriate dose calculation algorithms especially in the case of lung tumors (e.g. with limited heterogeneity correction), which may lead to mis-estimation of the absorbed dose. Additionally, outcome of different tumor stages, dose levels and fractionation schemes are frequently reported together. Furthermore, including historical cohorts implies dealing with changes over time in the standard diagnostic and therapeutic procedures, e.g. for staging, recurrence assessment and radiotherapy image guidance.

To counteract this variability, we applied strict inclusion criteria to the selected publications. The analysis is based on 3-year local control in order to depict the dose-response in mature outcome data, while maintaining a sufficient number of data points. To our knowledge this is one of the largest, most homogeneous patient collectives among similar studies. Through the combination of conventionally fractionated and hypofractionated data, a broad range of doses and fractionation schemes is covered, which is a further requirement in order to achieve conclusive modeling results.

Dosimetric heterogeneity in the PTV can be very pronounced in dose distributions for stereotactic treatments, reaching dose differences between isocenter and PTV edge of up to 50 %. It is not possible to know a priori, which reported dosimetric parameter will describe best the dose-effect relationship: the dose at the PTV edge or the dose at isocenter. Therefore, we calculated models based on both, isocenter and minimum target doses. The TCD_50_ doses estimated for the isocenter doses were in general higher than the ones from the models at PTV edge.

Variability in the estimated doses at PTV edge could arise for instance, from variations in the CTV and PTV margin definitions among institutions, or uncertainties in the dose calculation methods, which in the case of outdated, less accurate dose calculation algorithms for the lung, would produce large dose mis-estimation and underdosage at the PTV edge.

We calculated the Spearman’s correlations between outcome parameters and the BED_10_ evaluated at both dose specification points and observed that the correlations with BED_10_ at the PTV edge were without exception lower than the corresponding correlations based on BED_10_ at the isocenter. This could be interpreted as an indication of the isocenter doses being more robust than the doses at the PTV edge for retrospective modeling studies, in agreement with previous studies [61].

The BED_10_ fits based on the CF data and the complete dataset differ in both the TCD_50_ and k values. This can be explained by the fact that the information required defining the slope of the dose-response curve and the TCD_50_, which together shape the sigmoid region of the logistic function, is provided by the CF data, where generally a lower BED was applied. This explains why TCD_50_ and k are not consistently determined across models, which use different input data: CF + HF, or HF/CF alone. We represented the range of the model input data and the extrapolation regions in all figures, to stress that special care must be taken predicting doses in the extrapolation region.

For comparison, Fig. 1 includes the previously published dose-response models of Martel et al. [28] and Guckenberger et al. [61]. Martel et al. found a TCD_50_, which is higher as compared to our findings as they compiled stage I/II data whereas our dataset included stage I tumors only. The values for the slope γ_50_ that we obtained for the complete dataset and the CF subset are lower than the value in Martel et al. [28] and further values in the literature. For instance, Stuschke et al. [11] reported a value of 1.5, and Okunieff et al. [26] found a value of 1.6 on average.

Our fit based on the HF data alone shows a shallower curve than the fits including CF data, since the outcomes after hypofractionation are well above the inflection point of the sigmoidal curve. Our HF fit results for both BED_10_(isocenter) and BED_10_(PTV edge) are graphically similar to the findings by Guckenberger et al. [61], which were based on a multicentric compilation of individual patient SBRT data.

The LQ-L model was proposed to account for the experimental observation that curves of the (log cell survival) versus radiation dose often show a more straightened portion at doses beyond 10 Gy than predicted by the LQ model. This effect is in the majority of cases due to heterogeneity of the radiosensitivity distribution of the cells. This is partly due to differences in the stage of cells in the proliferation cycle, but can also be due to partially hypoxic conditions. Resistant cells tend to survive even at larger doses, which causes the survival curve to become less steep than predicted by the LQ model. This explanation implies that the straightening in the curve is not caused by a fundamental mechanism but by a simple to explain heterogeneity in the distribution of sensitivity. Both models converge at dose of less than about 6 Gy.

Applying state-of-the-art fitting methods to compare the performance of the LQ versus the LQ-L models for different fractionation schemes, we did not find significant differences. Therefore, it was not possible to decide, which model better predicts clinical NSCLC outcome data. For BED corresponding to doses per fraction below 11.0 Gy, the BED points for both concepts overlap, whereas above D_t_ there is a contraction in the BED_LQ-L_ values, which has no consequence in the fit itself, since it takes place in the region where the TCP approaches 100 %. Clinical consequences of using one model or another are only relevant for highly hypofractionated schedules aiming at delivering BED values well above 100 Gy.

The LQ-L model was also fitted to the HF datasets alone, although we could not obtain a 68 % CI for the TCD_50_, and the *p* values of all three parameters were > 0.05. Fig. 2b clearly demonstrates the similarity of our LQ-L fit for the HF data subset to the LQ fit previously presented, therefore TCP predictions will be similar with either model. Although our LQ-L fit based on HF data did not yield significant estimates, the results suggest a D_t_ estimate in the same range of magnitude of 10 Gy. This fit was tested previously also on hypofractionated data alone by Guckenberger et al. [61]. Their dataset had a median dose per fraction at isocenter of 20.8 Gy with range [6–41] Gy. This group found a D_t_ value of 22 Gy with a broad 68 % confidence interval, [14–42] Gy, whose addition to the model did not improve the prediction power.

Estimation of the α/β ratio by fitting three parameters simultaneously on a clinical dataset presenting high dispersion - as is the case of the current work - is challenging. Our LQ model with free α/β did not yield significant values for α/β, nor for TCD_50_ and the slope k, which appears to be too shallow after visual inspection. We concluded that there is no indication for larger α/β values than 10 Gy if the complete range of fractionations is considered. The opposite trend (α/β > 10 Gy) was found for the HF dataset as was also the case in Chi et al. [15], although no significant *p* values could be obtained in this case, neither.

There are few works aiming to the estimation of a clinical α/β for radiotherapy of NSCLC [10, 11, 15]. Thames et al. [10] published an extremely high α/β value for lung tumors but these authors did not obtain a reliable confidence interval and so, their calculations must be regarded with caution. A similar work was also carried out by Stuschke et al. [11], who found an α/β value of 8.2 Gy. They used a fit similar to ours, but set a constraint to force the model to approach the axes origin by adding a point with low BED and null TCP, with a high fit weight. We also tested this approach (full model information in the Additional file 1), adding a data point at 0 Gy (BED_α/β_) and 0 % TCP. We observed that this constraint influenced the TCD_50_ value to a small extent only, but could have a strong effect on the slope of the curve, and also on the α/β value, for instance, 3.9 [2.2–9.0] versus 12.6 [10.5–15.0] Gy for the complete dataset and BED_10_(isocenter). The LQ-based fits with α/β of 10 Gy for the CF data alone did not vary much with and without constraint. In contrast, if the fit was based on the HF dataset alone, adding this constraint on the original fit had a pronounced effect on the steepness and TCD_50_ of the curve, which in the constrained fits approached the curves based on CF data (Fig. 4a). For this reason, we think it is preferable not to set a constraint to the model, which largely influences the estimates for k and α/β, and also their standard errors. It seems reasonable to accept that fits done on the HF dataset alone will not reproduce a realistic fall-off in LC at low doses nor a plausible, clinical TCD_50_, since the input dose-response data are well above that region in this specific case.

The dispersion in the collected data points was high. Due to this fact, in the hypofractionation range the dose-effect relationship appears to be weaker than in other reports [13, 61]. Specifically, for the fraction of HF data above 100 Gy (BED_10_), the Spearman’s correlation between LC and BED_10_ is low. It can be speculated that in the region of high tumor control probability and highly hypofractionated treatments the relative contribution of non-radiobiological factors to the treatment effect is larger. Such factors are, for instance, subjectivity in target delineation and geographical miss, among others.

The logistic fit was applied also in the study at high dose regions since it is widely used for dose-effect description. However, other functional dependencies of LC with BED might be also appropriate in the high BED range. Concerning the BED concept, it seems likely that more than one approach can fit equivalently well on an inherently noisy dataset like this, for instance the LQ applying a higher α/β ratio, or the LQ-L model with α/β of 10 Gy, even if these models have contrasting radiobiological implications.

## Conclusion

We found a dose-effect relationship in the studied dataset, which in the high BED region was weaker due to considerable dispersion in the data. Both, the LQ and LQ-L models can be fitted to clinical normo- and hypofractionated NSCLC outcome data. The LQ-L model yielded a significant value for the D_t_ of 11.0 Gy for the model based on BED_10_(isocenter); however, it produced a comparable TCP fit to the LQ model. For the application of BED_10_ in the range of 100–150 Gy in three fractions or more, the differences in isoeffects predicted by both models can be neglected. Our findings therefore do not allow us to suggest use of the LQ-L model for an improved fitting compared to the LQ model of local control data in case of hypofractionation. A tentative analysis to establish the optimal α/β ratio in the frame of the LQ model for the full fractionation range did not produce significant estimates, although, it showed a trend for α/β values lower than 10 Gy.

## Additional file

Additional file 1: Details of the Pubmed search and of the studies included in this work. (PDF 197 kb)

## References

[1] Barendsen GW (1982). Dose fractionation, dose rate and iso-effect relationships for normal tissue responses. Int J Radiat Oncol Biol Phys.

[2] Joiner MC, van der Kogel AJ (2009). Basic clinical radiobiology.

[3] Herrmann T, Baumann M, Dörr W (2006). Klinische Strahlenbiologie -- kurz und bündig.

[4] Bentzen SM, Dörr W, Gahbauer R, Howell RW, Joiner MC, Jones B (2012). Bioeffect modeling and equieffective dose concepts in radiation oncology -- terminology, quantities and units. Radiother Oncol.

[5] Guckenberger M, Andratschke N, Alheit H, Holy R, Moustakis C, Nestle U (2014). Definition of stereotactic body radiotherapy: principles and practice for the treatment of stage I non-small cell lung cancer. Strahlenther Onkol.

[6] Guckenberger M, Allgauer M, Appold S, Dieckmann K, Ernst I, Ganswindt U (2013). Safety and efficacy of stereotactic body radiotherapy for stage I non-small-cell lung cancer in routine clinical practice: a patterns-of-care and outcome analysis. J Thorac Oncol.

[7] Fowler JF (2006). Development of radiobiology for oncology -- a personal view. Phys Med Biol.

[8] Kirkpatrick JP, Brenner DJ, Orton CG (2009). Point/counterpoint. the linear-quadratic model is inappropriate to model high dose per fraction effects in radiosurgery. Med Phys.

[9] Brown JM, Carlson DJ, Brenner DJ (2014). The tumor radiobiology of SRS and SBRT: are more than the 5 Rs involved?. Int J Radiat Oncol Biol Phys.

[10] Thames HD, Bentzen SM, Turesson I, Overgaard M, Van den Bogaert W (1990). Time-dose factors in radiotherapy: a review of the human data. Radiother Oncol.

[11] Stuschke M, Pottgen C (2010). Altered fractionation schemes in radiotherapy. Front Radiat Ther Oncol.

[12] Zhang J, Yang F, Li B, Li H, Liu J, Huang W (2011). Which is the optimal biologically effective dose of stereotactic body radiotherapy for stage I non-small-cell lung cancer? a meta-analysis. Int J Radiat Oncol Biol Phys.

[13] Mehta N, King CR, Agazaryan N, Steinberg M, Hua A, Lee P (2012). Stereotactic body radiation therapy and 3-dimensional conformal radiotherapy for stage I non-small cell lung cancer: a pooled analysis of biological equivalent dose and local control. Pract Radiat Oncol.

[14] van Baardwijk A, Bosmans G, Bentzen SM, Boersma L, Dekker A, Wanders R (2008). Radiation dose prescription for non-small-cell lung cancer according to normal tissue dose constraints: an in silico clinical trial. Int J Radiat Oncol Biol Phys.

[15] Chi A, Wen S, Liao Z, Fowler J, Xu J, Nguyen NP (2013). What would be the most appropriate alpha/beta ratio in the setting of stereotactic body radiation therapy for early stage non-small cell lung cancer. Biomed Res Int.

[16] Guerrero M, Li XA (2004). Extending the linear-quadratic model for large fraction doses pertinent to stereotactic radiotherapy. Phys Med Biol.

[17] Laine AM, Pompos A, Timmerman R, Jiang S, Story MD, Pistenmaa D, Choy H (2016). The role of hypofractionated radiation therapy with photons, protons, and heavy ions for treating extracranial lesions. Front Oncol.

[18] Bush DA, Cheek G, Zaheer S, Wallen J, Mirshahidi H, Katerelos A (2013). High-dose hypofractionated proton beam radiation therapy is safe and effective for central and peripheral early-stage non-small cell lung cancer: results of a 12-year experience at Loma Linda University Medical Center. Int J Radiat Oncol Biol Phys.

[19] Nihei K, Ogino T, Ishikura S, Nishimura H (2006). High-dose proton beam therapy for stage I non-small-cell lung cancer. Int J Radiat Oncol Biol Phys.

[20] Hata M, Tokuuye K, Kagei K, Sugahara S, Nakayama H, Fukumitsu N (2007). Hypofractionated high-dose proton beam therapy for stage I non-small-cell lung cancer: preliminary results of a phase I/II clinical study. Int J Radiat Oncol Biol Phys.

[21] Krämer M, Scholz M (2000). Treatment planning for heavy-ion radiotherapy: calculation and optimization of biologically effective dose. Phys Med Biol.

[22] Astrahan M (2008). Some implications of linear-quadratic-linear radiation dose-response with regard to hypofractionation. Med Phys.

[23] Park C, Papiez L, Zhang S, Story M, Timmerman RD (2008). Universal survival curve and single fraction equivalent dose: useful tools in understanding potency of ablative radiotherapy. Int J Radiat Oncol Biol Phys.

[24] Wang JZ, Huang Z, Lo SS, Yuh WTC, Mayr NA (2010). A generalized linear-quadratic model for radiosurgery, stereotactic body radiation therapy, and high-dose rate brachytherapy. Sci Transl Med.

[25] Moher D, Liberati A, Tetzlaff J, Altman DG (2009). Preferred reporting items for systematic reviews and meta-analyses: the PRISMA statement. BMJ.

[26] Okunieff P, Morgan D, Niemierko A, Suit HD (1995). Radiation dose-response of human tumors. Int J Radiat Oncol Biol Phys.

[27] Bentzen SM, Tucker SL (1997). Quantifying the position and steepness of radiation dose-response curves. Int J Radiat Biol.

[28] Martel MK, Ten Haken RK, Hazuka MB, Kessler ML, Strawderman M, Turrisi AT (1999). Estimation of tumor control probability model parameters from 3-D dose distributions of non-small cell lung cancer patients. Lung Cancer.

[29] R version 2.15.0, 2012-03-30, Copyright (C) 2012, The R Foundation for Statistical Computing, ISBN 3-900051-07-0.

[30] Kaskowitz L, Graham MV, Emami B, Halverson KJ, Rush C (1993). Radiation therapy alone for stage I non-small cell lung cancer. Int J Radiat Oncol Biol Phys.

[31] Jeremic B, Shibamoto Y, Acimovic L, Milisavljevic S (1997). Hyperfractionated radiotherapy alone for clinical stage I nonsmall cell lung cancer. Int J Radiat Oncol Biol Phys.

[32] Hayakawa K, Mitsuhashi N, Saito Y, Nakayama Y, Furuta M, Sakurai H (1999). Limited field irradiation for medically inoperable patients with peripheral stage I non-small cell lung cancer. Lung Cancer.

[33] Cheung PCF, Yeung LTF, Basrur V, Ung YC, Balogh J, Danjoux CE (2002). Accelerated hypofractionation for early-stage non-small-cell lung cancer. Int J Radiat Oncol Biol Phys.

[34] Langendijk JA, Aaronson NK, de Jong JMA, ten Velde GPM, Muller MJ, Slotman BJ (2002). Quality of life after curative radiotherapy in stage I non-small-cell lung cancer. Int J Radiat Oncol Biol Phys.

[35] Bradley JD, Wahab S, Lockett MA, Perez CA, Purdy JA (2003). Elective nodal failures are uncommon in medically inoperable patients with stage I non-small-cell lung carcinoma treated with limited radiotherapy fields. Int J Radiat Oncol Biol Phys.

[36] Bogart JA, Alpert TE, Kilpatrick MC, Keshler BL, Pohar SS, Shah H (2005). Dose-intensive thoracic radiation therapy for patients at high risk with early-stage non-small-cell lung cancer. Clin Lung Cancer.

[37] Zehentmayr F, Wurstbauer K, Deutschmann H, Fussl C, Kopp P, Dagn K (2015). DART-bid: dose-differentiated accelerated radiation therapy, 1.8 Gy twice daily: high local control in early stage (I/II) non-small-cell lung cancer. Strahlenther Onkol.

[38] Onimaru R, Shirato H, Shimizu S, Kitamura K, Xu B, Fukumoto S (2003). Tolerance of organs at risk in small-volume, hypofractionated, image-guided radiotherapy for primary and metastatic lung cancers. Int J Radiat Oncol Biol Phys.

[39] Xia T, Li H, Sun Q, Wang Y, Fan N, Yu Y (2006). Promising clinical outcome of stereotactic body radiation therapy for patients with inoperable stage I/II non-small-cell lung cancer. Int J Radiat Oncol Biol Phys.

[40] Fritz P, Kraus HJ, Blaschke T, MAhlnickel W, Strauch K, Engel-Riedel W (2008). Stereotactic, high single-dose irradiation of stage I non-small cell lung cancer (NSCLC) using four-dimensional CT scans for treatment planning. Lung Cancer.

[41] Onimaru R, Fujino M, Yamazaki K, Onodera Y, Taguchi H, Katoh N (2008). Steep dose-response relationship for stage I non-small-cell lung cancer using hypofractionated high-dose irradiation by real-time tumor-tracking radiotherapy. Int J Radiat Oncol Biol Phys.

[42] Baumann P, Nyman J, Hoyer M, Wennberg B, Gagliardi G, Lax I (2009). Outcome in a prospective phase II trial of medically inoperable stage I non-small-cell lung cancer patients treated with stereotactic body radiotherapy. J Clin Oncol.

[43] Brown WT, Wu X, Fayad F, Fowler JF, García S, Monterroso MI (2009). Application of robotic stereotactic radiotherapy to peripheral stage I non-small cell lung cancer with curative intent. Clin Oncol (R Coll Radiol).

[44] Fakiris AJ, McGarry RC, Yiannoutsos CT, Papiez L, Williams M, Henderson MA (2009). Stereotactic body radiation therapy for early-stage non-small-cell lung carcinoma: four-year results of a prospective phase II study. Int J Radiat Oncol Biol Phys.

[45] Kopek N, Paludan M, Petersen J, Hansen AT, Grau C, Hoyer M (2009). Co-morbidity index predicts for mortality after stereotactic body radiotherapy for medically inoperable early-stage non-small cell lung cancer. Radiother Oncol.

[46] Stephans KL, Djemil T, Reddy CA, Gajdos SM, Kolar M, Mason D (2009). A comparison of two stereotactic body radiation fractionation schedules for medically inoperable stage I non-small cell lung cancer: the Cleveland clinic experience. J Thorac Oncol.

[47] Baba F, Shibamoto Y, Ogino H, Murata R, Sugie C, Iwata H (2010). Clinical outcomes of stereotactic body radiotherapy for stage I non-small cell lung cancer using different doses depending on tumor size. Radiat Oncol.

[48] Crabtree TD, Denlinger CE, Meyers BF, El Naqa I, Zoole J, Krupnick AS (2010). Stereotactic body radiation therapy versus surgical resection for stage I non-small cell lung cancer. J Thorac Cardiovasc Surg.

[49] Timmerman R, Paulus R, Galvin J, Michalski J, Straube W, Bradley J (2010). Stereotactic body radiation therapy for inoperable early stage lung cancer. JAMA.

[50] Videtic GMM, Stephans K, Reddy C, Gajdos S, Kolar M, Clouser E (2010). Intensity-modulated radiotherapy-based stereotactic body radiotherapy for medically inoperable early-stage lung cancer: excellent local control. Int J Radiat Oncol Biol Phys.

[51] Andratschke N, Zimmermann F, Boehm E, Schill S, Schoenknecht C, Thamm R (2011). Stereotactic radiotherapy of histologically proven inoperable stage I non-small cell lung cancer: patterns of failure. Radiother Oncol.

[52] Hamamoto Y, Kataoka M, Yamashita M, Nogami N, Sugawara Y, Kozuki T (2012). Factors affecting the local control of stereotactic body radiotherapy for lung tumors including primary lung cancer and metastatic lung tumors. Jpn J Radiol.

[53] Lagerwaard FJ, Verstegen NE, Haasbeek CJA, Slotman BJ, Paul MA, Smit EF (2012). Outcomes of stereotactic ablative radiotherapy in patients with potentially operable stage I non-small cell lung cancer. Int J Radiat Oncol Biol Phys.

[54] Shibamoto Y, Hashizume C, Baba F, Ayakawa S, Manabe Y, Nagai A (2012). Stereotactic body radiotherapy using a radiobiology-based regimen for stage I non-small cell lung cancer: a multicenter study. Cancer.

[55] Shirata Y, Jingu K, Koto M, Kubozono M, Takeda K, Sugawara T (2012). Prognostic factors for local control of stage I non-small cell lung cancer in stereotactic radiotherapy: a retrospective analysis. Radiat Oncol.

[56] Takeda A, Kunieda E, Sanuki N, Aoki Y, Oku Y, Handa H (2012). Stereotactic body radiotherapy (SBRT) for solitary pulmonary nodules clinically diagnosed as lung cancer with no pathological confirmation: comparison with non-small-cell lung cancer. Lung Cancer.

[57] Inoue T, Katoh N, Onimaru R, Shimizu S, Tsuchiya K, Suzuki R (2013). Stereotactic body radiotherapy using gated radiotherapy with real-time tumor-tracking for stage I non-small cell lung cancer. Radiat Oncol.

[58] Takeda A, Sanuki N, Eriguchi T, Kaneko T, Morita S, Handa H (2013). Stereotactic ablative body radiation therapy for octogenarians with non-small cell lung cancer. Int J Radiat Oncol Biol Phys.

[59] Hamaji M, Chen F, Matsuo Y, Kawaguchi A, Morita S, Ueki N (2015). Video-assisted thoracoscopic lobectomy versus stereotactic radiotherapy for stage I lung cancer. Ann Thorac Surg.

[60] Rwigema JC, Chen AM, Wang PC, Lee JM, Garon E, Lee P (2014). Incidental mediastinal dose does not explain low mediastinal node recurrence rates in patients with early-stage NSCLC treated with stereotactic body radiotherapy. Clin Lung Cancer.

[61] Guckenberger M, Klement RJ, Allgauer M, Appold S, Dieckmann K, Ernst I (2013). Applicability of the linear-quadratic formalism for modeling local tumor control probability in high dose per fraction stereotactic body radiotherapy for early stage non-small cell lung cancer. Radiother Oncol.

