# Supplementary material for: Challenges in radiobiological modeling: can we decide between LQ and LQ-L models based on reviewed clinical NSCLC treatment outcome data?
Source: Radiat Oncol. 2016 May 6;11:67. doi: 10.1186/s13014-016-0643-5 (PMC4859978; doi:10.1186/s13014-016-0643-5)
Supplement: Additional file 1: — Details of the Pubmed search and of the studies included in this work. (PDF 197 kb) [file 13014_2016_643_MOESM1_ESM.pdf]

Details of the search made in PubMed (July 2015):

(early stage[Title] OR early-stage[Title] OR stage I[Title] OR stage II[Title]) AND (non-small cell lung cancer[Title] OR non-small-cell lung cancer[Title] OR non-small cell lung cancer[Title] OR NSCLC[Title] OR lung cancer[Title] OR lung tumors[Title] OR lung tumor[Title] OR lung carcinoma[Title]) AND (radiother*[Title] OR SBRT[Title] OR stereotactic[Title] OR irradiation[Title] OR radiation[Title])

Number of records identified through database searching: 441
Number of records after duplicates removed: 441
Number of records screened: 441
Number of records excluded: 441-192=249
Number of full-text articles assessed for eligibility: 192
Number of full-text articles excluded, with reasons: 192-31=161 did not fulfill the selection criteria
Number of additional records identified through other sources: Four files found: Hamamoto12 , Onimaru03 , Takeda12 , Takeda13.
Number of studies included in this quantitative analysis: 31 studies, 8 3DCRT + 23 SBRT.

Table 1: Detailed characteristics of included studies with conventionally fractionated treatment schedules

Reference	Tumor Stage	No. Pats	Histology	Loca tion	Schedule, Total Treatment Time Prescription	Total Dose at isocenter [Gy]	Volume Definition	LC [%]	CSS [%]	OS [%]	FU median (range) [m]
Kaskowitz	stage IA/IB 20 T1 - 33 T2, N0	53	11 ade, 32 scc, 4 lc, 6 nos	ns	median 63 (40 - 80) Gy, conv. fractionation, dose point not specified	63§	target: tumor + 1.5 cm (estimated from port films), elective nodal RT	3y: 51	3y: 33 5y: 13	3y: 19 5y: 6	ns
Jeremic	stage IA/IB 25 T1 - 24 T2, N0	49	18 ade, 23 scc, 7 lc, 1 nos	25 p 24 c	69.6 Gy in 1.2 Gy/f (twice a day), 40 days, to isocenter	70	PTV: tumor + ipsilateral hilus + 2 cm	3y: 55 5y: 55		3y: 47 5y: 30	ns
Hayakawa	stage IA/IB 7 T1 – 29 T2, N0	36	16 ade, 19 scc, 1 lc	p	60 - 81 Gy in 2 Gy/f, 48 days, to isocenter	67§	target not specified, 28 % patients elective nodal RT to 38 - 50 Gy	3y: 72 5y: 72	3y: 56 5y: 39	3y: 42 5y: 23	(36 - 216)
Cheung	stage IA/IB 18 T1 - 15 T2, N0	33	10 ade, 13 scc, 5 lc, 5 nos	27 p 6 c	48 Gy in 4 Gy/f, maximum 3 weeks, to isocenter	48	target: GTV + 1 - 1.5 cm, no elective nodal RT	3y: 63	3y: 36	3y: 32	23&
Langendijk	stage IA/IB 26 T1 - 20 T2, N0	46	2 ade, 23 scc, 10 undiff, 11 unknown	37 p 9 c	70 Gy in 2 Gy/f, 7 weeks to isocenter, Dmin(PTV)= 90 %	70	CTV: GTV + 1.5 cm; PTV_{46Gy}: CTV + 0.5 cm, PTV_{70Gy}: GTV + 1 cm; elective nodal RT	3y: 50		3y: 22	36&
Bradley	stage IA/IB 31 T1 - 25 T2, N0	56	14 ade, 25 scc, 6 lc, 11 nos	p	60 - 84 Gy in 1.8 - 2 Gy/f, 6 - 8 weeks to isocenter	70$	PTV: GTV + 1 cm (increased if tumor moved) 39 % patients elective nodal RT	3y: 63	3y: 51	3y: 34	20 (6 - 72)
Bogart	stage IA/IB 19 T1 - 12 T2, N0	31	9 ade, 8 scc, 1 lc, 13 nos	ns	median 70 Gy in 2.25 - 3.7 Gy/f, 5.5 weeks, 95 % dose covering PTV	70$	PTV: GTV + 1 - 1.5 cm (increased if tumor moved), no elective nodal RT	3y: 83 5y: 83		3y: 64 5y: 19	29&
Zehentmayr	stage I	40 +14 stage II	16 ade, 33 scc, 7 nos* 14 stage II reported together, but not in LC	36 p 2 c	Median 79.2 (73.8-90.4) Gy in 1.8 Gy/f twice daily in 2-3 weeks, dose point not specified	79	Slow CT, PTV: GTV + 7mm	3y: 91			28.5 (2-108)
**Median**		**43**				**70**		**3y: 63 5y: 72**	**3y: 43.5**	**3y: 34 5y: 21**	**28.5 (2 - 216)**

FU: follow-up, §: mean value, $: median value, ade: adenocarcinoma, scc: squamous cell carcinoma, ba: bronchio-alveolar, lc: large cell carcinoma, nos: not otherwise specified, undiff: undifferenciated, non-scc: non-squamous cell carcinoma, ns: not specified, &: pats alive at the end of follow up, p: peripheral, c: central.
Kaskowitz: 54% of total patients received total doses between 60 and 70 Gy, with a median difference in total treatment time of 5 days, the maximal difference was 28 days. Hayakawa: Standard deviation of 7 Gy in the total dose and 8 days in total treatment time.
Cheung: included 4 patients with N1. Bradley: A majority of patients (42%) were receiving between 70 and 83 Gy. Bogart: Total doses range from 60 to 80 Gy, "most frequently 70 Gy" (sic).

Table 2: Detailed summary of hypofractionated data.

Reference	Tumor Stage	No Pats	Histology	Loca tion	Schedule, Total Treatment Time Prescription	Total Dose at isocenter [Gy]	Volume definition	LC [%]	CSS [%]	OS [%]	FU median (range) [m]
Onimaru '03	stage IA/IB 17 T1 - 8 T2, N0	25	14 ade, 8 scc, 3 nos	p	48 Gy in 6 Gy/f or 60 Gy in 7.5/f, 2 weeks, to isocenter, Dmin(PTV) = 80 %	48[$]	CTV = GTV, ITV: CTV in free breathing, exhale, and inhale, PTV: ITV+ 0.5 cm	3y: 55	2y: 60	2y: 47	18 (2-44)[#]
Xia	stage IA/IB 25 (T1 + T2), N0	25	all with pathological confirmation	p, c	50 Gy in 5 Gy/f in 2 weeks 50 % isodose to PTV edge	100	CTV = GTV PTV: GTV + 1 cm	3y: 96		3y: 91	27 (24 - 54)
Fritz	stage IA/IB 22 T1 - 18 T2, N0	40	ade 17, scc 8, lc 13, nos 2	p	30 Gy in 1 single fraction, to isocenter, to cover 80 % of PTV, 90 % of GTV	30	CTV: GTV, ITV: CTV, mid-cycle, inhale, exhale, PTV: ITV + 1 cm axial, 1.5 cm cc	3y: 81	3y: 57	3y: 66	20 (6 - 62)
Onimaru '08	stage IA/IB 13 T1 - 28 T2, N0	41	30 ade, 10 scc, 1 lc	p	40 Gy in 10 Gy/f or 48 Gy in 12Gy/f, 1 week, to isocenter, Dmin(PTV) = 80 %	48[$]	CTV = GTV (CT end exhale)+6-8 mm, PTV: CTV+ 0.5 cm	3y: 57	3y: 53	3y: 47	27[&] (9 - 62)
Baumann	stage IA/IB 40 T1 - 17 T2, N0	57	19 ade, 8 scc, 1 lc, 10 nos, 19 ns	ns	45 Gy in 15 Gy/f, in median 5 (4-15) days to 67 % isodose to PTV edge	66	CTV: GTV +2mm PTV: CTV + 0.5-1.0cm axial, 1 cm cc	3y: 92	3y: 88	3y: 60	35 (4 - 47)
Brown	stage IA/IB 20 T1 - 11 T2, N0	31	8 ade, 1 scc, 1 ba, 21 nos	p	60 - 67.5 Gy in 3 or 5 fractions, prescribed to the 60-80% isodose line	88[$]	CTV: GTV + 0.6 cm, PTV: CTV + 0.2 cm, tumor tracking mit CyberKnife	3y: 86		3y: 84	28 (24 - 53)
Fakiris	stage IA/IB 34 T1 - 36 T2, N0	70	ns	22 c 48 p	60 Gy in 20 Gy/f or 66 Gy in 22Gy/f, to 80 % isodose at PTV edge	83[$]	CTV = GTV PTV: CTV + 0.5-1 cm axial, 1 cm cc	3y: 88	3y: 82 5y: 70	3y: 43 5y: 17	50 (1 - 65)
Kopek	stage IA/IB 51 T1 - 36 T2, N0	88	30 ade, 34 scc, 24 nos	62 p 26 c	45 Gy in 15 Gy/f or 67.5 Gy in 22.5 Gy/f to isocenter in 5-8 days, minD(PTV)=67%	45[$]	CTV = GTV PTV: CTV + 0.5 cm axial and 1 cm cc	3y: 89	3y: 70	3y: 37	44 (2 - 97)
Stephans	stage IA/IB 42 T1 - 14 T2, N0	56	9 ade, 20 scc, 7 undiff/other, 20 ns	p, c	50 Gy in 10 Gy/f in 11 (8 - 14) days, 97 - 100 % isodose to PTV egde	70	ITV: GTV in free breathing, inhale, ex-hale, PTV: ITV + 0.5 cm axial + 1 cm cc	3y: 97		3y: 50	20 (2 - 48)
Baba	T1 87, T2 37	124	66 ade, 35 scc, 13 nos, 10 unproven	nn	48/52 Gy in 12/13 Gy/f in 11 (8 – 14) days, to isocenter, Dmin(95% PTV) =80%	48[$]	CTV = GTV, ITV: CTV in 3 breathing phases, PTV: ITV + 0.5 cm axial, 1 cm cc	3y: 80			26 (7 - 66)
Crabtree	stage IA/IB 57 T1 - 19 T2, N0	76	ns	ns	54 Gy in 18 Gy/f, in 8 to 14 days, to the 80 % to 85 % isodose line	68	ns	3y: 89	3y: 67	3y: 32	19
Timmerman	stage IA/IB 44 T1 - 11 T2, N0	55	19 ade, 17 scc, 16 nos, 3 lc undiff	p	54 Gy in 18 Gy/f in maximum 2 weeks 100 % isodose to PTV edge	79	CTV = GTV PTV: CTV + 0.5 cm axial, 1 cm cc	3y: 98	3y: 55	3y: 48	34 (5 - 50)
Videtic	stage IA/IB 22 T1 - 6 T2, N0	26	13 ade, 4 scc, 3 nos, 8 ns	25 p 3 c	50 Gy in 10 Gy/f, 5 days, PTV enclosed by 95 % isodose line, IMRT planning	54	ITV: GTV in free breathing, exhale, and inhale, PTV: ITV(=CTV) + 0.3-0.5 cm	3y: 94		3y: 52	31 (10 - 51)
Andratschke	stage IA/IB 31 T1 - 61 T2,	92	35 ade, 49 scc, 2 ba, 6 nos	24 c 68 p	24-45 Gy in 3-5 fractions in 5-12 days, to 60 % isodose to PTV edge	62[$]	CTV = GTV, ITV: CTV in slow CT PTV: ITV + 0.5 cm axial and 1 cm cc	3y: 83 5y: 83	3y: 64 5y: 48	3y: 38 5y: 17	21 (3 - 87)

	N0										
Hamamoto	stage IA/IB 101 T1 - 27 T2, N0	128	ns	ns	48 or 60 Gy in 9.2-14 Gy/f ,in 4 to 10 days, to isocenter, Dmin(PTV) = 90 %	48[$]	ITV: GTV with slow CT, PTV: ITV + 0.5 cm	3y: 85			18 (1 - 60)
Lagerwaard	stage IA/IB 106 T1 - 71 T2, N0	177	20 ade, 16 scc, 24 nos, 117 ns	p, c	5 × 12 Gy, 3 × 20 Gy, or 8 × 7.5 Gy in 2 weeks, to 80 % isodose at PTV edge	75[$]	CTV = GTV, ITV: GTV from 10 resp. phases, PTV: ITV + 3mm	3y: 93<br>5y: 93		3y: 85<br>5y: 51	32
Shibamoto	T1 - T2		104 ade, 60 scc, 16 nos	35 c<br>145 p	dosage depended on size, T1: 48 Gy, T2: 52 Gy		CTV = GTV, ITV: CTV in 3 resp. phases PTV: ITV + 0.5 cm axial + 1 cm cc	3y: 83<br>5y: 83	3y: 83<br>5y: 69	3y: 69<br>5y: 52	36 (42[&])
Shibamoto d2	124 T1, N0	124			48 Gy in 12 Gy/f, in 9 to 21 days, 95 % of the ITV > 94 % of presc. dose	48		3y: 86			
Shibamoto d3	52 T2, N0	52			52 Gy in 13 Gy/f, in 9 to 21 days, 95 % of the ITV > 94 % of prescribed dose	52		3y: 73			
Shirata	stage IA/IB 63 T1 - 18 T2, N0	80	33 ade, 22 scc, 5 lc, 20 nos		prescription to isocenter, Dmin(PTV) = 90 %		CTV: GTV + 0-0.5 cm, PTV: CTV + 0.5-1 cm, individualized margins	3y: 89	3y: 97	3y: 90	30 (0.3 - 79)
Shirata, d1		45			48 Gy in 12 Gy/f	48		3y: 100			
Shirata, d2		29			60 Gy in 7.5 Gy/f	60		3y: 82			
Takeda			ade 64, scc 38, nos 13, ns 58	p, c	in one week, to 80 % isodose at PTV edge		ITV: GTV in slow CT (6-8 s/slice) PTV: ITV + 0.6-0.8 cm (indiv. margins)				
Takeda, d1	27 (10 T1 + 17 T2)~	27			40 Gy in 8 Gy/f	50		3y: 72			21 (6 - 64)
Takeda, d2	138 (91 T1 + 47 T2)~	138			50 Gy in 10 Gy/f	63		3y: 87			21 (6 - 64)
Inoue	stage IA/IB 79 T1 - 30 T2, N0	109	65 ade, 29 scc, 1 lc, 8 nos, 6 ns	ns	45 Gy in 15 Gy/f or 48 Gy in 12 Gy/f in 4 to 7 days, to isocenter	48[$]	CTV: GTV + 5 – 8mm PTV: CTV + 5mm	3y: 81<br>5y: 78		3y: 69<br>5y: 64	25 (4 - 72)
Takeda	stage IA/IB 67 T1 - 42 T2, N0	109	41 ade, 13 scc, 8 nos, 47 ns	34 c<br>75 p	40 or 50 Gy in 8 or 10 Gy/f, in 5 days, to 80 % isodose at PTV edge	63[$]	ITV: GTV in slow CT PTV: ITV + 0.6-0.9 cm	3y: 84.4	3y: 71	3y: 54	24 (3 - 65)
Hamaji	75 T1, 29 T2	104	ade: 54, scc: 34, large cell ca 4, others 0, nos 12	nn	48 Gy in 12 Gy/f in 5 days, to isocenter	48	ITV= GTV w/ slow CT/4DCT, PTV= ITV + 5mm	3y: 76.7			43 (6 - 115)
Rwigema	40 Ia, 6 Ib	46	ade 35, scc 4, adenosquamous 2, nos: 5	41 p<br>5 c	54 Gy in 18 Gy/f in 5 days, to PTV edge	70	4DCT, ITV-MIP 8 phases, PTV=ITV+3mm transv+6mm in long direction	3y: 95.5			16.8 (0.6 – 38.9)
**Median**		**57**				**57.0**		**3y: 85.9<br>5y: 83**	**3y: 70<br>5y: 69**	**3y: 54<br>5y: 51**	**27 (0.3 - 115)**

FU: follow-up, §: mean value, $: median value, ade: adenocarcinoma, scc: squamous cell carcinoma, ba: bronchialveolar, lc: large cell carcinoma, nos: not otherwise specified, undiff: undifferenciated, non-scc: non-squamous cell carcinoma, ns: not specified, #: includes the patients with metastases, &: patients alive at the end of follow-up, p: peripheral, c: central.

Onimaru03: local control for the NSCLC group only, 12 Gy difference in total dose among patients, 17 patients got 48 Gy versus 8 patients who got 60 Gy in total with the same number of

fractions.
Onimaru08: 8 Gy difference in total dose among patients, 13 patients received 40 Gy and 28 patients 48 Gy both with four fractions.
Baba: 8 Gy difference in total dose among patients. All 30 stage IA lesions were treated with 48 Gy, and 12 stage IB lesions were treated with 52 Gy, all in 4 fractions.
Xia: stage I pats 25/43.
Brown: 26 out of 31 patients received 60 Gy, most frequently in 3 fractions (24/31).
Kopek: plus one patient T3 N0. Total dose to isocenter was 45 Gy for 62/88 patients, or 67.5 Gy for 26/88 patients who beared peripheral lesions, all delivered in three fractions.
Andratschke: large cohort with most tumors receiving total doses of 24 - 45 Gy in 3 - 5 fractions.
Hamamoto: large cohort of 128 NSCLC patients, the majority received 48 Gy in 4 fractions.
Lagerwaard: 177 patients in total, 82/177 (46%) got 12 Gy per fraction. Maximal difference in the number of fractions is 5.
Takeda: proportion T1-T2 tumors, via personal communication with the authors.

Table 3: Summary of the models for biologically effective doses calculated at isocenter and PTV edge with a constraint to make the logistic curves approach the coordinate origin; all fit parameter values are provided with standard errors (and 68% CI).

Model concept and dataset		α/β (std error) [Gy]	D_t (std error) [Gy]	TCD_{50} CI 68% [Gy]	k CI 68% [Gy]	γ_{50} (std error) [%/%]	AIC
			**ISOCENTER**				
**LQ with constraint at (0,0)**	**CF + HF**	**α/β = 10**	-	72.6 (68.2-74.9)*	22.8 (20.1-25.5)*	0.79(0.11)	-4773.8
	**CF**	**α/β = 10**	-	71.2 (67-74.9)*	16.4 (12.0-20.8)*	1.08(0.36)	-267.1
	**HF**	**α/β = 10**	-	75.9 (71.0-81.0)*	21.5 (17.9-24.8)*	0.88(0.17)	-2875.8
**LQ: free α/β with constraint at (0,0)**	**CF + HF**	12.6(10.5-15.0)*	-	67.1(62.4-71.7)*	19.3(15.7-23.0)*	0.87(0.17)	-4780.1
	CF	3.9(2.4-6.6)	-	90.8(77.4-110.0)*	18.3(12.7-27.0)	1.24(0.59)	-270.1
			**PTV EDGE**				
**LQ with constraint at (0,0)**	**CF + HF**	**α/β = 10**	-	54.6 (51.5-57.6)*	18.3 (16.1-20.6)*	0.74(0.12)	-4761.9
	**CF**	**α/β = 10**	-	66.5 (62.7-69.9)*	15.5 (11.6-19.5)*	1.07(0.33)	-266.8
	**HF**	**α/β = 10**	-	51.5 (48.3-54.7)*	17.2 (15.1-17.2)*	0.75(0.12)	-5294.6
**LQ: free α/β with constraint at (0,0)**	**CF + HF**	5.8(4.7-7.1)*	-	69.7(63.1-77.0)*	23.2(19.6-27.5)	0.75(0.16)	-4764.7
	CF	4.2(2.4-8.0)	-	81.3(68.8-99.4)	17.7(12.5-25.5)	1.15(0.62)	-267.8

* p value < 0.05

Table 4: Doses per fraction at isocenter and PTV edge, calculated according to the information provided in the references (prescription in bold characters).

Reference	d @ Isocenter	d @ PTV edge	Comment
**Conventionally fractionated treatment schedules**			
Kaskowitz 1993	**1.8**	1.7	If no minimal dose to the PTV was explicitly reported, it was assumed to be 95% dose, according to ICRU recommendations. This was done for all the conventional treatments. Ratios deviate from 0.95 because of rounding error.
Jeremic 1997	**1.2**	1.1	
Hayakawa 1999	**2**	1.9	
Cheung 2002	**4**	3.8	
Langendijk 2002	**2**	1.9	
Bradley 2003	**1.9**	1.8	
Bogart 2005	**2.5**	2.4	
Zehentmayr 2015	**1.8**	1.7	
**Hypofractionated treatment schedules**			
Onimaru 2003	**6**	4.8	The dose was prescribed at the isocenter, with the 80% line encompassing the PTV
Xia 2006	10	**5**	The 50% isodose line covered 100% PTV, therefore it was assumed that dose at isocenter was twice the prescription
Fritz 2008	**30**	24	The dose prescribed to the isocenter was 30 Gy. Of the prescribed isocenter dose, at least 90% covered the gross tumor volume (GTV = CTV) and at least 80% the PTV.
Onimaru 2008	**12**	9.6	The dose was prescribed at the center of the PTV, aim was the inclusion of the PTV in the 80% isodose
Baumann 2009	22	**15**	The patients were treated with a dose of 15 Gy times three at about the 67% isodose to the periphery of the PTV, resulting in a central dose of about 22 Gy x 3. More specifically the mean of the maximum dose/fraction to PTV was 22.8 Gy (SD, 3.1 Gy) and the average value of the mean dose/fraction to CTV was 21.6 Gy (SD, 3.0 Gy).
Brown 2009	29.4	**20**	A 60-67.5 Gy dose was prescribed to the 60-80% isodose line (median 65%) and given in three to five fractions
Fakiris 2009	27.5	**22**	The treatment dose was prescribed to the 80% isodose volume
Kopek 2009	**15**	10.1	The prescription dose (45 Gy or 67.5 Gy in three fractions) was delivered to the isocenter. The CTV was encompassed by the 95% isodose surface while the PTV was completely covered by the 67% isodose surface. This corresponds to a minimum dose to the PTV of 30 Gy or 45 Gy in three fractions, depending on the central prescribed dose.
Stephans 2009	14	**10**	Patients treated to 60 Gy were typically planned using three or more dynamic arcs without heterogeneity corrections prescribed to the 81 to 90% isodose line (as allowed by RTOG 0236).
Baba 2010	**12**	9.6	The dose was prescribed at the isocenter; 95% of the PTV was ensured to be covered with at least 80% of the prescribed isocenter dose.
Crabtree 2010	22.5	**18**	The dose is typically prescribed to the 80% to 85% isodose line, meaning that the center of the tumor received a dose that is 15% to 20% higher than the prescription.
Timmerman 2010	26.3	**18**	Edge of the PTV, 95% of PTV received 100% of prescribed dose; from Xiao and Papiez related publication: isocenter dose ranges from 71.3 Gy to 88.9 Gy (mean 78.8 Gy, SE 1.1 Gy, ie 26.3 Gy per fraction). This was a s study based on a subset of 20 patients of the RTOG 0236 trial.

Videtic 2010	10.8	**10**	Prescription: minimum dose in median to the PTV was 9.9Gy (~10Gy), and max dose in median was 10.8Gy. It was assumed that the dose at the isocenter was similar to the maximum dose inside the PTV (stage I, small tumor volumes).
Andratschke 2011	20.8	**12.5**	Doses were prescribed to the 60% isodose covering the planning target volume (PTV)
Hamamoto 2012	**12**	10.8	Leaf margins were arranged so that the 90–95 % isodose line covered the PTV. The dose calculation algorithm was the pencil beam method; this algorithm did not use heterogeneity correction. In SBRT, 48–60 Gy in 4–5 fractions was delivered to the isocenter
Lagerwaard 2012	15	**12**	All fractionation schemes used were prescribed to the encompassing 80% isodose
Shibamoto d2	**12**	9.6	The prescribed dose represented the dose delivered to the isocenter. It was recommended to cover 95% of the PTV with at least 90% of the isocenter dose; in all patients, 95% of the PTV received at least 80% of the prescribed dose. Consequently, 95% of the ITV was covered with !94% of the prescribed dose in all but 1 patient.
Shibamoto d3	**13**	10.4	
Shirata 2012 d1	**12**	10.8	The target reference point was defined as the center of the PTV, and the dose was prescribed for its point. PTV was encompassed by the minimum 90% dose line of the reference point dose as possible.
Shirata 2012 d2	**7.5**	6.8	
Takeda 2012 d1	**10**	8	The prescribed dose was defined as 80% of the maximal dose and its isodose line encompassed the PTV surface. Then the median D95 was consistent with the prescribed dose. For peripheral tumors, a total of 50 Gy/5 fractions/ 5 days was prescribed. For tumors adjacent to critical organs such as trachea, main bronchus, pulmonary artery and esophagus, the total dose was decreased to 40 Gy/5 fractions/ 5 days.
Takeda 2012 d2	**12.5**	10	
Inoue 2013	**12**	9.6	Using a superposition algorithm, they administered 48 Gy in 4 fractions at the isocenter in 2005–2006 (n = 30) and 40 Gy in 4 fractions to the 95% volume of PTV in 2007–2010 (n = 79) with a treatment period of 4 to 7 days. Isocenter dose of 40 Gy in 4 fractions to the 95% volume of PTV was approximately ranged from 45 to 50 Gy. Therefore it was used 12 Gy at isocenter and 9.6 Gy (80%) at edge. Good approximation (min dose 38.4 Gy)
Takeda 2013	12.5	**10**	80% isodose of the maximum dose at PTV periphery
Hamaji 2015	**12**	9.6	Prescription to the isocenter, it was not specified which isodose line surrounds the PTV. It was assumed the 80%.
Rwigema 2015	23.4	**18**	To PTV edge, coverage at 95% of PTV normalized to prescription dose. Heterogeneity in PTV of 15-40%

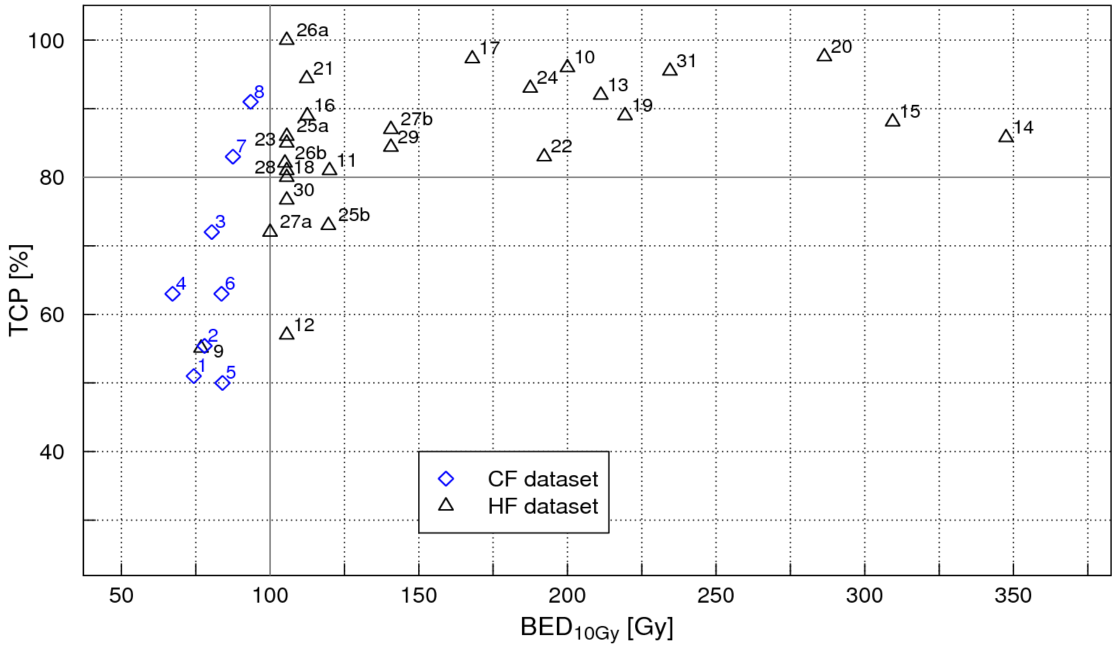

Figure 1: Local control of the conventionally fractionated dataset (blue) and hypofractionated dataset (black) versus BED_{10} calculated at the isocenter, with reference numbers.
